# Influenza Colds

**Published:** 1892-02

**Authors:** 


### INFLUENZA COLDS.

"Few remedies are more reliable, and act better as a preventive, or lessen the distressing symptoms of an influenza cold than the following mixture :

R Sodii salicylas, dram jss.
Liq. ammon. acet., oz. ij.
Aq. camph., ad oz. vj.

Misce. Capt. : oz ss. omnis 3tiis horis.

If this be taken every two or three hours when the first symptoms of cold come on, it will usually ward off the attack."—*British and Colonial Druggist.*

